# Whole-Blood Testing for Diagnosis of Acute Zika Virus Infections in Routine Diagnostic Setting

**DOI:** 10.3201/eid2507.182000

**Published:** 2019-07

**Authors:** Jolanda J.C. Voermans, Suzan D. Pas,, Anne van der Linden, Corine GeurtsvanKessel, Marion Koopmans, Annemiek van der Eijk, Chantal B.E.M. Reusken

**Affiliations:** Erasmus Medical Center, Rotterdam, the Netherlands

**Keywords:** Zika virus, whole blood, RT-PCR, diagnostics, viruses, diagnosis

## Abstract

We evaluated the benefit of whole blood versus plasma to detect acute Zika virus infections. Comparison of Zika virus quantitative reverse transcription PCR results in single timepoint whole blood–plasma pairs from 227 patients with suspected Zika virus infection resulted in confirmation of 8 additional patients with Zika virus infection.

Since its emergence in South and Central America in 2015/2016, Zika virus (genus *Flavivirus*) has become a major public health concern. Zika virus infections are linked to congenital malformations in neonates from mothers infected during pregnancy and to neurologic disorders in adults (*1*). Thus, the stakes for an accurate diagnosis are high when congenital Zika syndrome might be involved, such as in diagnosis in pregnant women and their partners, because Zika virus infections can be sexually transmitted (*1*). Diagnostics are based on Zika virus RNA detection, detection of Zika virus–specific antibodies, or both. However, a definitive diagnosis based on serology only is hampered by the existence of a high degree of cross-reactivity between Zika virus and other flaviviruses and their vaccines. In addition, populations with a high background of other flavivirus infections, such as dengue virus, might lack high-titer Zika virus–specific antibody production (also known as original antigenic sin) (*2*,*3*). Reverse transcription PCR (RT-PCR) is the most reliable method for confirming Zika virus infections. Viremia in pregnant women can be prolonged, up to 70 days, but more commonly the window of detection for Zika virus RNA in serum or plasma is much shorter (3–14 days after onset of symptoms). The window of detection can be considerably longer for urine and semen, but these specimens are not routinely collected (*4*–*7*).

Various studies have suggested that flavivirus genomic RNA might be detectable for longer periods in whole blood than in plasma, thereby expanding the timeframe for viral genome detection to up to 120 days after onset of symptoms (*4*,*5*,*8*–*11*). Therefore, molecular detection of Zika virus RNA in whole blood instead of plasma might improve Zika virus case confirmation (*12*,*13*). In a prospective study, we systematically evaluated the benefit of whole blood versus plasma as the sample of choice to detect acute Zika virus infections in a routine diagnostic setting.

## The Study

We compared Zika virus quantitative reverse transcription PCR (qRT-PCR) results for 249 EDTA–whole blood and EDTA–plasma pairs submitted for laboratory testing from 227 patients with suspected Zika virus infection during July 2016–May 2017. These patients were those with a Zika virus diagnostic request in this period from whom both plasma and whole blood could be collected. In line with previous observations in our laboratory (*14*), the first day of illness was provided infrequently, in only 29 (12.8%) of the 227 patients.

Using a standard EDTA blood collection tube, we aliquoted 600 μL of whole blood before the centrifugation step (10 min at 2,400 × *g*) to collect plasma. We stored the samples at −80°C until use. For testing, we spiked the samples with an internal control and extracted total nucleic acids from a 500-μL sample in 100 μL of eluate using the MagNAPure 96 DNA and Viral NA large volume kit and Viral NA Universal LV 2.0 protocol (Roche, https://www.roche.com*),* according to the manufacturer’s instructions. Extraction was followed by an ISO15189:2012-validated laboratory-developed Zika virus qRT-PCR, as described previously (*15*). We confirmed all Zika virus RNA−positive samples using a commercial Zika virus qRT-PCR (Altona Diagnostics, http://www.altona-diagnostics.com), as described by the manufacturer. 

We detected Zika virus RNA in 31 (12.4%) of 249 whole-blood samples and in 23 (74.2%) of the 31 corresponding plasma samples. The 31 positive whole-blood samples were collected from 31 individual patients. This comparison indicated that 8 additional Zika virus–positive patients would have been identified if whole blood had been used routinely instead of plasma (Figure). This finding represented a 34% increase in confirmed cases of Zika virus infection.

**Figure:**
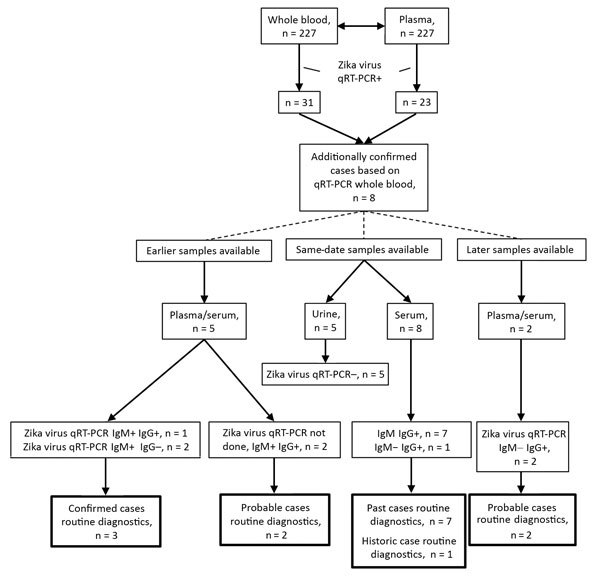
Overview of results of Zika virus diagnostic testing on total sample sets for 8 Zika patients additionally confirmed with Zika virus infection on the basis of whole-blood qRT-PCR. IgG, Zika virus IgG ELISA; IgM, Zika virus IgM ELISA; qRT-PCR, quantitative reverse transcription PCR; +, positive; –, negative.

Standard practice in international guidelines on diagnostic algorithms for Zika virus is to combine molecular testing of plasma with molecular testing of urine, along with serology, to come to an accurate Zika virus diagnosis. However, confirmation of cases based on serology only is usually limited to expert Biosafety Level 3 laboratories being able to perform virus neutralization tests comparing Zika virus titers with titers of other flaviviruses (*12*,*13*). In our center, we routinely perform qRT-PCR on plasma and urine while running ELISA IgM/IgG testing in parallel on corresponding serum samples, provided these samples are submitted by treating physicians. Preferably, ELISAs are performed on paired serum samples taken at least 2 weeks apart (acute and convalescent phases) to monitor titer changes. However, these paired samples are not always submitted; for example, in our study cohort a second sample was provided for only 11 (61.1%) of 18 patients who were seropositive by ELISA and RT-PCR negative in plasma in the initial sample.

To determine whether our routine Zika virus testing algorithm, in which whole blood is not a sample of choice, would have missed the 8 additional identified patients, we evaluated the Zika virus test results of the complete sample set submitted for these patients (Figure). We tested urine and plasma by qRT-PCR as described previously and tested serum by ELISA (Euroimmun, https://www.euroimmun.com) for the presence of Zika virus–specific IgM and IgG, as described by the manufacturer. For 3 of the 8 additional patients, Zika virus infection had already been confirmed on the basis of the presence of Zika virus RNA and IgM in an earlier plasma sample. For the remaining 5 patients, only a status of probable case was achieved without the whole-blood testing (*12*). Two of these patients had a status of probable infection on the basis of the presence of Zika virus IgM and IgG in an earlier sample, but no PCR was performed. Seven patients had the status of a probable Zika virus infection on the basis of serology performed on a same-date serum sample, and 1 patient had the status of past infection because of the absence of IgM. Two patients had no evidence for a recent Zika virus infection on the basis of a later serum sample that tested negative for RNA IgM and positive for IgG. The semiquantitative Zika virus ELISA did not show significant titer changes between different collection dates (data not shown).

## Conclusions

Our overall results indicate that, in our routine diagnostic algorithm in the absence of whole-blood testing, the infections of 5 of 227 patients would have been identified as probable Zika virus cases, whereas with whole-blood testing, they would have been identified as confirmed cases on the basis of positive qRT-PCR results. In cases for which only 1 sampling date would have been available, our systematic analysis showed that, of infections in 227 patients, 8 additional Zika virus cases would have been confirmed. Based on these observations, we conclude that individual patient care might benefit from whole-blood testing in a routine diagnostic laboratory setting, thereby possibly reducing the need for more specialized serology (i.e., comparative flavivirus neutralization tests) to confirm cases based on serology. Therefore, we have implemented whole-blood RT-PCR testing for Zika virus diagnostic requests in our routine diagnostic setup. Further studies in larger cohorts, including dengue and chikungunya virus testing, as well to address the often multiplex settings in endemic countries, are needed to demonstrate the general usefulness of our observations.
